# Needs assessment for improving library support for dentistry researchers

**DOI:** 10.5195/jmla.2019.556

**Published:** 2019-07-01

**Authors:** Helen Yueping He, Madeline Gerbig, Sabrina Kirby

**Affiliations:** Head of Dentistry Library, University of Toronto, Toronto, ON, Canada, helen.he@dentistry.utoronto.ca; Instruction & Liaison Librarian, Dentistry Library, University of Toronto, Toronto, ON, Canada, maddy.gerbig@mail.utoronto.ca; Intern, Toronto Academic Libraries (TALint Program), Dentistry Library, University of Toronto, Toronto, ON, Canada, sabrina.kirby@mail.utoronto.ca

## Abstract

**Objective:**

To better support dentistry researchers in the ever-changing landscape of scholarly research, academic librarians need to redefine their roles and discover new ways to be involved at each stage of the research cycle. A needs assessment survey was conducted to evaluate faculty members’ research support needs and allow a more targeted approach to the development of research services in an academic health sciences library.

**Methods:**

The anonymous, web-based survey was distributed via email to full-time researchers at the Faculty of Dentistry, University of Toronto. The survey included twenty questions inquiring about researchers’ needs and behaviors across three stages of the research cycle: funding and grant applications, publication and dissemination, and research impact assessment. Data were also collected on researchers’ use of grey literature to identify whether current library efforts to support researchers should be improved in this area.

**Results:**

Among library services, researchers considered support for funding and grant applications most valuable and grey literature support least valuable. Researcher engagement with open access publishing models was low, and few participants had self-archived their publications in the university’s institutional repository. Participants reported low interest in altmetrics, and few used online tools to promote or share their research results.

**Conclusions:**

Findings indicate that increased efforts should be made to promote and develop services for funding and grant applications. New services are needed to assist researchers in maximizing their research impact and to increase researcher awareness of the benefits of open access publishing models, self-archiving, and altmetrics.

## INTRODUCTION

The landscape of scholarly communications has changed rapidly in recent years. As a result, academic libraries have had to adapt in an effort to expand their role in supporting and contributing to the research activities at their institutions [1–3]. Increasingly, academic libraries are aligning their services to the strategic visions of their home institutions to continue growing and evolving in their supportive roles [3, 4]. Maxwell argues that the rise in importance of research activity on university campuses creates an opportune channel through which the library can engage with institutional objectives [3].

Many recent studies, such as those conducted by Harp Ziegenfuss and Furse [5] and Haddow and Mamtora [6], demonstrate that librarians are working diligently to explore and assess new ways of meeting researchers’ needs. As faculty still place the highest importance on the library’s traditional collections-based role [7–9], librarians cannot assume that researchers know about the variety of research services that are offered in libraries. In addition, many researchers may lack awareness of librarians’ skills and potential contributions to the research process [1, 2, 8, 10]. Some researchers question librarians’ qualifications and competencies, which may lead them to undervalue or fail to fully take advantage of library support services [11–14]. Improved communication [10, 15, 16] and targeted marketing and outreach efforts [1–3, 5] are vital to creating successful research support services in academic libraries. However, it is equally important for librarians to develop a better understanding of researcher needs and perspectives [1–3].

New roles for librarians are emerging in response to shifts and innovations in the contemporary research landscape, especially due to new technologies [11–13, 17–20]. Librarians are called upon to move beyond their comfort zones and develop new skills to keep up with researchers’ evolving needs [1, 2, 5, 6, 11–14, 17, 18, 21–27]. In particular, studies indicate growing interest in and importance of social networking tools in academia, often as a means to improve reputations and increase collaborations. However, researchers vary greatly in their awareness, skills, use, and perceptions of these tools [28–34]. Both Persson and Svenningsson [35] and Tran and Lyon [33] recommend that librarians evaluate current knowledge and usage of relevant digital technologies among the faculty they serve to better design support services for researchers.

Given the strategic commitment of the Faculty of Dentistry, University of Toronto, to promote research to “shape and grow our research enterprise around targeted research foci” [36], and as the library only employs two librarians and one library technician, the authors wished to identify the services that our users felt were most beneficial. We could then arrange our limited resources efficiently to be more proactive in supporting faculty research—from idea generation through dissemination of findings. To this end, we aimed to find answers to the following questions:

1. What are current behaviors among researchers at the Faculty of Dentistry in the following areas: funding and grant applications, publication and dissemination, research impact assessment, and grey literature searching?
2. How do researchers currently use technology to assist in their activities at each of the stages identified above?
3. Where do dentistry faculty researchers believe there is the greatest need for library support services?

## METHODS

Using the research cycle proposed by Maxwell as a framework [3], we collected information about the needs of dentistry researchers at the Faculty of Dentistry, University of Toronto, during three of the six research stages: funding and grant applications, publication and dissemination, and research impact assessment. We chose these three stages of the research cycle to examine in the survey due to the high volume of questions that were historically received from faculty in these areas and that we, therefore, identified as having the highest relevance to the activities of Dentistry Library users.

In addition, these areas are also underrepresented in the literature. Although data management is an important aspect of the research life cycle, we did not select it for examination as other campus librarians are already conducting investigations in this area. The study also investigated researchers’ use of grey literature. This information was obtained to determine how users rank the value of grey literature to establish whether additional effort should be invested in recreating and/or updating the Dentistry Library’s Grey Literature & Statistics research guide.

With these questions in mind, we developed a web-based survey with twenty questions using SurveyWizard. The complete survey instrument is provided in the supplemental appendix. The survey asked participants to indicate what tools, resources, and strategies they used during each of the three identified stages of the research cycle. Participants were also asked to rank potential support services that the library could offer, based on their perceived usefulness. Library services listed in the survey were identified and chosen for inclusion based on findings from literature that were examined during a preliminary literature review. In addition, the survey included five questions about participants’ perceptions and use of grey literature for their research.

For the purposes of this survey, a dentistry researcher was defined as any faculty member (including adjunct and part-time) who conducted research. As not all faculty conducted research, not all faculty were considered researchers.

Prior to survey distribution, the research protocol was approved by the University of Toronto Health Sciences Research Ethics Board. An email invitation was sent on November 21, 2017, to all researchers at the Faculty of Dentistry via the Dentistry Faculty email discussion list to solicit participation in the survey. Since this list includes members who are not researchers, when counting the number of researchers, we printed out the list of members to exclude lecturers, status-only faculty, and some administrative staff. On the same date, an announcement of the study with a link to the survey was also posted on the library website. The survey was posted for four weeks, with a reminder sent one week before the survey closed. Participation in the survey was voluntary and involved no incentives. All survey responses were completely anonymous and confidential.

When analyzing data, for questions in which participants were asked to rank research services, the “average weighted score,” as described by SurveyMonkey, was calculated for each option to determine the overall order of preference for the options provided. The most preferred choice was given the largest weight, with the highest possible score equaling the number of options provided in the question. Where *W*=weight of ranked position and *X*=response count for answer choice, the formula provided by SurveyMonkey [37] for calculating average weighted score was:

$$\frac{X_{1}W_{1}+X_{2}W_{2}+\ldots X_{n}W_{n}}{\text{Total Response Count}}$$

This method was chosen as it gave a higher average to more important services and, thus, was more descriptive than “average rank” [37].

## RESULTS

We received a total of 15 responses to the survey. As there were 75 researchers at the Faculty of Dentistry at the time of survey distribution, we thus had a 20% overall response rate. Participants were generally senior researchers: 47% reported having 11–20 years of research experience and 40% reported having more than 20 years of research experience. For the question, “Which of the following best describes your primary research interest?,” we grouped the results into 2 main categories: clinical research (i.e., respondents who selected “clinical research”) and basic science research (i.e., all other respondents who did not select “clinical research”). Of all respondents, 13% worked in a clinical research area, while 87% indicated the basic sciences (e.g., biomaterials, biomedical engineering, microbiology) as their primary area of study. Among the 75 researchers in the Faculty of Dentistry, 43% were focused on clinical and 57% were focused on basic science. Therefore, the response rate for basic science researchers (30%) was much higher than that for clinical researchers (6%).

The survey included a preliminary set of questions to gauge participants’ overall interest in library workshops. Among the 67% of respondents who indicated that they were interested in library workshops, 60% preferred in-person workshops, 20% preferred webinars, and 10% preferred videos. The reasons given by the 33% of participants who were not interested in library workshops were: lack of time (n=2), most information is available online (n=1); and they were already familiar with all the tools needed (n=1).

Table 1 summarizes the rankings for the four research service categories we examined and the service rankings within these four discrete areas.

**Table 1:** Research service category rankings

Research service category	Average weighted score
Information services for funding and grant applications	3.07
Research dissemination assistance	2.60
Research impact assessment assistance	2.20
Grey literature searching assistance	1.87
Funding and grant application service rankings	
Assistance in the form of a mentorship program with successful grant seekers	7.46
Provision of general funder policy guidelines	7.36
Grant and funding databases training	7.12
Assistance with preparing impact statements for funders	7.00
Literature search advice	6.21
Reference management support	6.00
Assistance with identifying research priorities/known uncertainties	6.00
Assistance with preparing data management plans for grant applications	5.85
Assistance with remaining current with research topics in your field	5.46
Provision of guidelines for writing and/or publication	4.92
Assistance with open access requirements for funders	4.31
Publication and dissemination service rankings	
Assistance with reference management	4.36
Assistance with copyright issues	4.20
Assistance with meeting funder mandates and/or requirements	4.14
Assistance with identifying publication venues	4.00
Assistance with negotiating licenses	3.86
Assistance with open access publication	3.79
Assistance with archiving publications	3.43
Research impact assessment service rankings	
Research metrics training	8.77
Assistance with identifying funding agencies	7.29
Citation analysis guidance	6.79
One-on-one consultations	6.57
Assistance with tenure and promotion profiles	6.07
Assistance with benchmarking at departmental and institutional level	6.07
Provision of research trend reports	5.71
Assistance with topical bibliometric analysis	5.38
Assistance with identifying collaborators	4.85
Altmetrics training	4.69
Altmetrics support	4.23

### Funding and grant applications

Similar to studies by Hollister and Schroeder [9] and Cain et al. [15], library support for funding and grant applications was ranked as very valuable by participants. From a list of 11 potential research services that the library could provide during the funding/grant application process, those ranked by participants as the most beneficial for their research were assistance in the form of a mentorship program with successful grant seekers, provision of general funder policy guidelines, and training on grant and funding databases. Tri-agency grants—which are grants provided by three major federal granting agencies that promote and support research, research training, and innovation in Canada (Canadian Institutes of Health Research [CIHR], the Natural Sciences and Engineering Research Council of Canada [NSERC], and the Social Sciences and Humanities Research Council of Canada [SSHRC])—were ranked as the number one funding resource by 53% of participants.

The survey also asked respondents to indicate which tools and/or resources they used when working on funding or grant applications. Among researcher profiling tools, the majority of researchers (73%) reported using Google Scholar, while only 33% of researchers reported using ORCID. Two researchers reported using all 4 researcher profiling tools listed in the survey (Google Scholar, ORCID, Scopus Author ID, and Researcher ID). The most popular collaboration tools among respondents were Google Drive and Dropbox, which were used by 80% and 60% of participants, respectively. The majority of dentistry researchers indicated that they used EndNote as their preferred reference management tool (87%).

### Research dissemination

When asked to rank which research services would benefit them most during the research dissemination process, participants ranked assistance with reference management, with copyright issues, and in meeting funder mandates and requirements as the highest.

**Figure 1:**
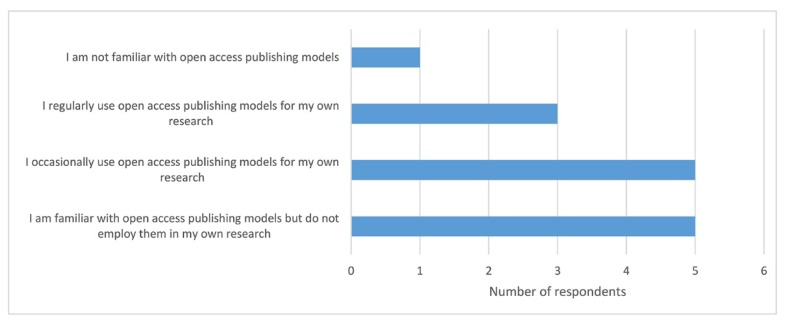
Open access engagement

Assistance with open access publication generated comparatively low interest among participants, which aligned with findings by Cain et al. [15]. While the majority of survey participants indicated that they were familiar with open access publishing models, only 20% regularly used these models to publish their own research (Figure 1).

When asked to indicate what they considered to be the most important factor when selecting a journal in which to publish, 43% of respondents considered the academic reputation of a journal to be an important factor when making publishing decisions, while only 7% (n=1) indicated the availability of open access to be important.

**Figure 2:**
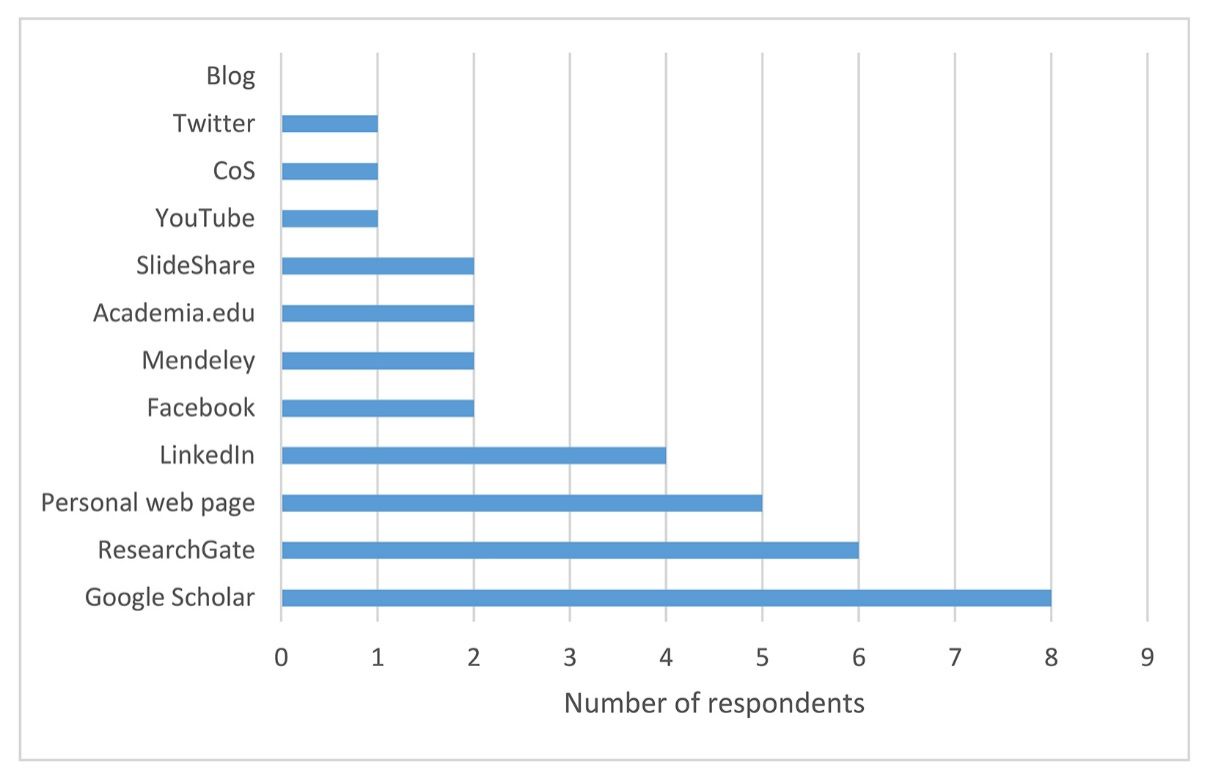
Social media platforms used for sharing research

Participants were also asked to indicate whether they had shared their research work online in nontraditional ways and, if so, which tools they used for this purpose. The most popular resources among survey respondents fell in the category of social media platforms. However, even the most popular platform, ResearchGate, was only used by 40% of researchers (Figure 2). A quarter (27%) of participants did not use any of the 15 social media platforms, researcher networks, and content sharing services listed.

### Research impact assessment

From a list of eleven research impact assessment services, those that were ranked as having the most potential value for participants’ research activities were research metrics training and assistance with identifying funding agencies. In comparison, altmetrics training and support services were ranked as having the least potential value.

**Figure 3:**
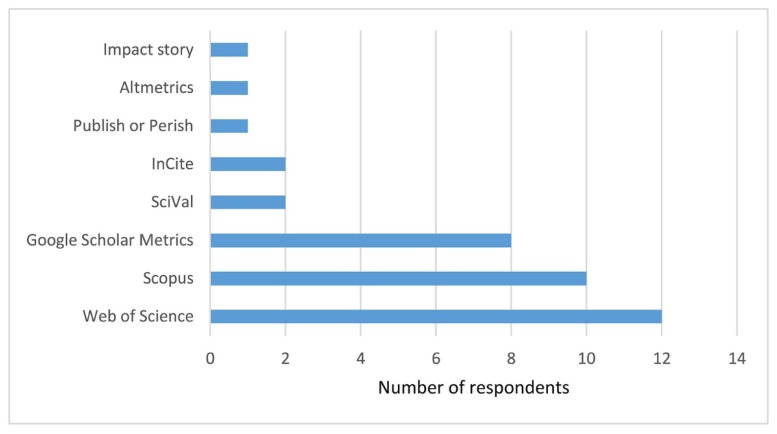
Resources for research impact assessment

When conducting research impact measurement activities (e.g., scholarly output, citation count, h-index), 80% of participants indicated that they used Web of Science (Figure 3). Other popular resources among participants were Scopus and Google Scholar Metrics, which were used by 67% and 53% of participants, respectively. Thirty-three percent of participants reported using all 3 of these resources.

When asked to rank which research services would benefit them the most for their research impact assessment activities, the majority of participants (62%) responded that support for research output metrics would be most beneficial.

**Figure 4:**
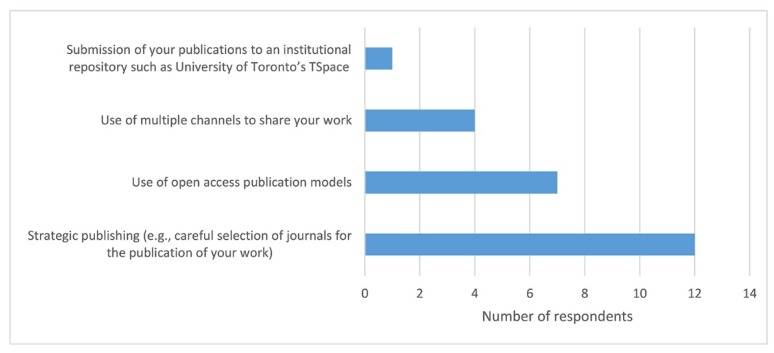
Strategies for maximizing research impact

Dentistry researchers use a combination of strategies to maximize their research impact (Figure 4). The method most commonly used by participants was strategic publishing (80%). Only 1 researcher reported publishing research in an institutional repository such as University of Toronto’s research repository, TSpace.

### Grey literature

Eighty percent of participants indicated that grey literature typically made up 1%–20% of the citations they used in their papers, while 20% of participants did not use any grey literature in their papers. Despite the majority of participants indicating that they used grey literature in their research, none considered grey literature to be “very important”: 60% of participants reported that it was “not important” or “not important at all.” Although grey literature searching assistance was ranked by participants as the least beneficial library service, only 21% of participants indicated that they found it easy or somewhat easy to search for and access grey literature.

**Figure 5:**
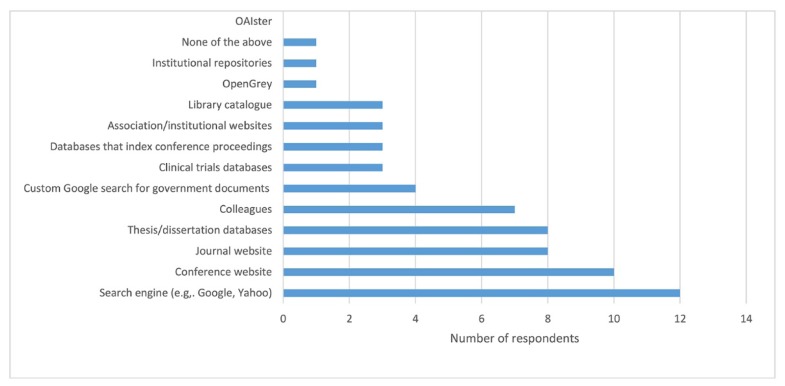
Resources for searching grey literature

Participants were also asked to rank the relative importance of five different types of grey literature for their research. The two grey literature resources that were considered most valuable by researchers were conference and seminar proceedings, followed by theses and dissertations. When asked to specify which tools they used when searching grey literature, the most popular resources were search engines, followed by conference websites (Figure 5).

## DISCUSSION

These findings provide an interesting snapshot of the information resources, tools, and services that dentistry researchers need and use at three stages of the research cycle. The results also help provide insight into faculty receptivity to grey literature in dentistry. Although the central library has a Scholarly Communications and Copyright Office with staff dedicated to support questions surrounding open access, self-archiving, and metrics, gaps emerged between researchers’ needs and the services that the library currently provides.

### Funding and grant applications

Although library support for funding and grant applications was ranked by participants as the most valuable form of assistance, the Dentistry Library does not currently offer the top four services that our researchers need.

Otter et al. pointed out that assistance with grant applications from library and information professionals “can save researchers’ time, provide specialist support, and contribute to reducing avoidable waste in research” [38]. Several studies demonstrate strategies for developing programs to support researchers’ funding and grant applications. These include collaboration with an institution’s research and innovation office to understand the services that it provides as well as navigation of the grant search process through the use of grant databases [2, 3, 39]. Librarians can also communicate with researchers to expand their role in the grant application process by supporting literature reviews as part of grant applications [40], coauthoring and becoming full participants on funded projects [12, 41], providing research metrics (i.e., bibliometrics) to help researchers support their grant applications [38, 40], and developing tools and resources to aid researchers with data management plans as part of grant applications [38].

### Publication and dissemination

While the majority of participants carefully selected journals for publication, most did not use any channels to share their research results post-publication, such as institutional repositories or social media platforms.

Among survey participants, submission of content for archiving in institutional repositories was very low. Only one had submitted their publications to TSpace, the university’s institutional repository. This result is similar to findings reported by Ukwoma and Mole [42]. Akpokodje and Akpokodje [43] listed a number of factors contributing to low use of institutional repositories, such as lack of awareness, knowledge, interest, and adequate technology, and/or other functional limitations. Further discussion with dentistry researchers is needed to determine the reasons for such low interest in self-archiving.

With the number of social media platforms continuing to increase, researchers have more opportunities to share their research with broader audiences and in nontraditional ways [44]. However, low engagement with social media platforms among participants raised questions as to how the library can offer support in this area. Some excellent examples from other academic libraries that we can learn from include:

1. Librarians at the University of Huddersfield in the United Kingdom instituted a pilot, voluntary Web 2.0 course for scholars to teach them how to leverage social networking tools for research activity and dissemination. New tools were introduced each week, encouraging participants to evaluate and experiment with web technologies in their professional lives [45].
2. Librarians at Linköping University in Sweden created a web-based information package, “Social Media and Networks for Researchers,” together with the university’s Communications and Marketing Division and Information and Communication Technologies studio. The package provides step-by-step guides to show researchers how to use social media to best distribute publications, gain visibility, and network and monitor their field [35].
3. Librarians at Stony Brook University, the State University of New York, investigated researchers’ use and awareness of unique author identifiers and their interest in library instruction programs focused on these and other digital technologies. As a result of their research, librarians offered training sessions in person and online that instructed researchers in creating profiles for “researcher networking systems” (i.e., ResearchGate) and introducing ORCID [33].

### Research impact assessment

When participants were asked to highlight the resources or tools that they used for conducting research impact measurement activities, only two researchers indicated that they were using SciVal and/or InCite. This result might indicate that many participants were not familiar with these two powerful research impact assessment tools that University of Toronto Libraries subscribed to. Alternatively, some researchers might know about these resources but considered more popular tools, such as Google Scholar Metrics and Web of Science, to be more efficient or easier to use.

It was also interesting to note that altmetrics support and training were ranked among the least valued research impact assessment services. One potential explanation was that most participants were senior researchers who might be less familiar with these new and emerging metrics and/or did not recognize the value of altmetric data for offering insights throughout the research life cycle. It was also possible that because these metrics were not required for tenure promotion or funding applications, researchers did not feel the need to learn about or use them.

Previous literature indicates that librarians’ participation in bibliometric analysis not only increases the visibility of the library [22, 46], but also builds librarians’ competencies [22]. As altmetrics complement traditional metrics, they have begun to become a part of the bibliometric services that librarians offer [47]. To generate researcher interest, Lapinski et al. have suggested adding altmetric perspectives to standard bibliometric instruction. One way of doing so is to offer workshops demonstrating the benefits of altmetrics for helping researchers track the engagement of their research in an online environment [48].

Since researchers and administrators can be drawn to a “simple” metric such as the h-index, the most effective use of bibliometric service will serve to educate researchers and encourage them to reflect in a productive way about their publication practices [49]. However, it is equally important to educate researchers about the limitations as well as benefits of the various research impact assessment tools and methods [47]. Some notable programs that have recently been offered in this area include the development of a platform to generate faculty research productivity profiles for analyzing and visualizing research impact metrics [50], creation of an online bibliometrics guide [46], and establishment of a research impact measurement service to provide reports involving the h-index (or variations), identify top publications, and analyze citations for researchers [51].

### Grey literature

Although grey literature support was identified as the least important service, the majority of participants indicated that they used grey literature in their research and few reported that they felt it was easy to find and access. A comprehensive research guide might help researchers with their grey literature research needs. As participants considered finding grey literature to be a low priority compared to other research tasks, our library chose an online guide as the most appropriate service option. Compared to workshops, one-on-one consultations, and other services, research guides require much less effort and time commitment from busy researchers.

## RECOMMENDATIONS

For researchers to realize all the benefits of what the library offers, communication between librarians and faculty is essential in providing research and reference support. More efforts should be made to develop marketing strategies to promote libraries’ existing and new research support services. Below are key recommendations and strategies that we plan to investigate as a result of the survey.

- Continue to deepen relationships with researchers by exploring ways of getting more involved in their research process, including drafting a “research impact statement” for funding applications and assisting with tri-agency open access policy compliance.
- Continue to promote existing services and resources by developing workshops or enriching research-support web pages pertaining to specific topics.

Marketing strategies being considered for promoting these services are:

- Offer a research tip in the Faculty of Dentistry’s monthly newsletter and tweet the tip through the library’s Twitter account.
- Dedicate a month to promoting research support services with a different theme each week. Offer drop-in sessions and workshops related to weekly themes. Employ special tweets or posters related to events and resources for each theme.
- Attend the research round organized by the research office.

### Limitations

The survey was created based on reviewed literature. Because articles on this topic are primarily written by librarians, the survey design and results may reflect librarians’ internal perceptions of library services and lack a full consideration of researchers’ perspectives. Another limitation is that this research only examines research support activities from the literature studied. To develop a comprehensive list of research support services, an environmental scan of those listed on other academic libraries’ websites could be conducted.

Most study participants had more than ten years of research experience, and the expectations of senior established researchers might well differ from those of less experienced ones. The majority of participants were also researchers in basic sciences disciplines; therefore, perspectives of clinical science researchers might be underrepresented by the results of the survey. Further research is necessary to provide a better understanding of how to improve library support for early career and clinical science researchers and to assess whether their needs differ from those of our study participants. This study also focused solely on dentistry faculty; and the suite of library support services that they desired might be different from that of researchers in other fields. Finally, since the survey was voluntary, the responses only included people who chose to participate and, therefore, might not be as reliable as conclusions based on a random sample of the entire population under consideration.

## CONCLUSIONS

This study improves understanding of dentistry researchers’ needs for funding and grant applications, publication and dissemination, and research impact assessment along with identifying the potential support services that the University of Toronto Dentistry Library could consider offering in the future. The results of this investigation will guide more meaningful and efficient research service development and delivery at the Dentistry Library, University of Toronto.

To better support researchers throughout the research cycle, librarians need to either expand their roles or be strategic in promoting the services they currently provide. We need to explore ways of adopting the next generation of digital tools and provide new services to enhance engagement, connectivity, and collaboration among academic researchers [15]. We must also keep in mind that researchers adopt information tools and services that are easy to use and simplify their work, even when those tools and services are not optimal, comprehensive, or on their university’s “approved” list [52].

Research support services tend to be more effective when they are implemented based on an identified need or in response to an established issue, rather than as a broad “one-size-fits-all” approach [8]. Librarians should try not to impose their values onto researchers, but rather observe what researchers are already doing, learn to understand the research process, and identify tools that will make researchers’ lives easier and more efficient [53].

As Cain et al. point out, “while health science librarians strive to remain attuned to the information demands of the organizations they serve, understanding the breadth and depth of resources and services needed by researchers has not always been as clear-cut” [15]. While the results of this survey cannot be generalized across all researcher populations, they suggest the needs and behaviors of health sciences researchers in academic settings. The authors believe that these findings can help other academic health sciences librarians to develop strategies and services for supporting researchers at their institutions.

## SUPPLEMENTAL FILES

Appendix Survey instrument

## References

[1] Jubb M, Atkinson J (2016). Libraries and the support of university research. Quality and the academic library: reviewing, assessing and enhancing service provision.

[2] Atkinson J, Atkinson J (2016). Academic libraries and research support: an overview. Quality and the academic library: reviewing, assessing and enhancing service provision.

[3] Maxwell D (2016). The research lifecycle as a strategic roadmap. J Libr Adm.

[4] Anderson R (2014). Being essential is not enough, part 2: peer to peer review. Libr J.

[5] Harp Ziegenfuss D, Furse C (2016). Opening up collaboration and partnership possibilities: re-valuing library resources, skill sets, and expertise. Digit Libr Perspect.

[6] Haddow G, Mamtora J (2017). Research support in Australian academic libraries: services, resources, and relationships. New Rev Acad Librariansh.

[7] Sewell C, Kingsley D (2017). Developing the 21st century academic librarian: the Research Support Ambassador Programme. New Rev Acad Librariansh.

[8] Brown JM, Tucker C (2013). Expanding library support of faculty research: exploring readiness. Portal Libr Acad.

[9] Hollister CV, Schroeder R (2015). The impact of library support on education faculty research productivity: an exploratory study. Behav Soc Sci Librar.

[10] Falciani-White N (2016). Understanding the “complexity of experience”: modeling faculty research practices. J Acad Librariansh.

[11] Mamtora J (2013). Transforming library research services: towards a collaborative partnership. Libr Manag.

[12] Janke R, Rush KL (2014). The academic librarian as co-investigator on an interprofessional primary research team: a case study. Health Inf Libr J.

[13] Cox AM, Verbaan E (2016). How academic librarians, IT staff, and research administrators perceive and relate to research. Libr Inf Sci Res.

[14] Blatchford B, Borwick C, Glen S, Hall B, Harding A, Hilliar MA, Oakley S, Peters J, Smith S, Summers L, Whitfield R, Williams S (2016). Librarians supporting research in Wales: collaborative staff development and capacity building. SCONUL Focus.

[15] Cain TJ, Cheek FM, Kupsco J, Hartel LJ, Getselman A (2016). Health sciences libraries forecasting information service trends for researchers: models applicable to all academic libraries. Coll Res Libr.

[16] Epstein SA, Rosasco RE (2015). Connecting faculty researchers to librarians via departmental associates. Ref Libr.

[17] Kennan MA, Corrall S, Afzal W (2014). “Making space” in practice and education: research support services in academic libraries. Libr Manag.

[18] Keller A (2015). Research support in Australian university libraries: an outsider view. Aust Acad Res Libr.

[19] Macdonald K (2015). Collaborative partnerships for library services: examples from a hospital library. J Hosp Librariansh.

[20] McRostie D (2016). The only constant is change: evolving the library support model for research at the University of Melbourne. Libr Manag.

[21] Greyson D, Surette S, Dennett L, Chatterley T (2013). “You’re just one of the group when you’re embedded”: report from a mixed-method investigation of the research-embedded health librarian experience. J Med Libr Assoc.

[22] Astrom F, Hansson J (2013). How implementation of bibliometric practice affects the role of academic libraries. J Librariansh Inf Sci.

[23] Drummond R (2014). RIMS revisited: the evolution of the research impact measurement service at UNSW Library. Aust Acad Res Libr.

[24] Coombs J, Thomas M, Rush N, Martin E (2017). A community of practice approach to delivering research support services in a post-92 higher education institution: a reflective case study. New Rev Acad Librariansh.

[25] Ginther C, Lackner K, Kaier C (2017). Publication services at the University Library Graz: a new venture, a new role. New Rev Acad Librariansh.

[26] Crum JA, Cooper ID (2013). Emerging roles for biomedical librarians: a survey of current practice, challenges, and changes. J Med Libr Assoc.

[27] Vaughan KT, Hayes BE, Lerner RC, McElfresh KR, Pavlech L, Romito D, Reeves LH, Morris EN (2013). Development of the research lifecycle model for library services. J Med Libr Assoc.

[28] McMahon TM, Powell JE, Hopkins M, Alcazar DA, Miller LE, Collins L, Mane KK (2012). Social awareness tools for science research. D-Lib.

[29] Van Noorden R (2014). Online collaboration: scientists and the social network. Nature.

[30] Dermentzi E, Papagiannidis S, Osorio Toro C, Yannopoulou N (2016). Academic engagement: differences between intention to adopt social networking sites and other online technologies. Comput Hum Behav.

[31] Jamali HR, Nicholas D, Herman E (2016). Scholarly reputation in the digital age and the role of emerging platforms and mechanisms. Res Eval.

[32] Sugimoto CR, Work S, Larivière V, Haustein S (2017). Scholarly use of social media and altmetrics: a review of the literature. J Assoc Inf Sci Technol.

[33] Tran CY, Lyon JA (2017). Faculty use of author identifiers and researcher networking tools. Coll Res Libr.

[34] Kjellberg S, Haider J, Sundin O (2016). Researchers’ use of social network sites: a scoping review. Libr Inf Sci Res.

[35] Persson S, Svenningsson M (2016). Librarians as advocates of social media for researchers: a social media project initiated by Linköping University Library, Sweden. New Rev Acad Librariansh.

[36] University of Toronto Faculty of Dentistry (2014). achieving impact through excellence: strategic plan 2014–2019.

[37] SurveyMonkey (2018). Help center: ranking question [Internet].

[38] Otter MLE, Wright JM, King NV (2017). Developing the librarians’ role in supporting grant applications and reducing waste in research: outcomes from a literature review and survey in the NIHR Research Design Service. New Rev Acad Librariansh.

[39] Exner N (2015). Building faculty relationships through research support. Issues Sci Technol Librariansh.

[40] Karasmanis S, Murphy F Emerging roles and collaborations in research support for academic health librarians.

[41] Ziegenfuss DHdzue, Furse C (2016). Opening up collaboration and partnership possibilities. Digit Libr Perspect.

[42] Ukwoma SC, Mole AJC (2017). Utilisation of institutional repositories for searching information sources, self-archiving and preservation of research publications in selected Nigerian universities. Afr J Libr Archiv Inf Sci.

[43] Akpokodje VN, Akpokodje ET (2015). Availability and utilization of institutional repositories as indicators to institutional web ranking. Eur J Comput Sci Inf Technol.

[44] Cox MF, Brunson PC, Sambamurthy N, Branch SE, Berdanier CGP Transformation of faculty dissemination practices via social media.

[45] Stone G, Collins E (2013). Engaging researchers with social media tools: 25 Research Things@Huddersfield. Ariadne.

[46] Bladek M (2014). Bibilometrics services and the academic library: meeting the emerging needs of the campus community. Coll Undergrad Libr.

[47] González-Fernández-Villavicencio N, Domínguez-Aroca MI, Calderón-Rehecho A, García-Hernández P (2015). What role do librarians play in altmetrics?. An de Doc.

[48] Lapinski S, Piwowar H, Priem J (2013). Riding the crest of the altmetrics wave: how librarians can help prepare faculty for the next generation of research impact metrics. Coll Res Libr News.

[49] Drummond R (2016). Reflection on: “RIMS: the research impact measurement service at the University of New South Wales.”. Aust Acad Res Libr.

[50] Braun S (2017). Supporting research impact metrics in academic libraries: a case study. Portal Libr Acad.

[51] Drummond R, Wartho R (2016). RIMS: the research impact measurement service at the University of New South Wales (reprinted from Aust Acad Res Libr. 2009;40:76–87). Aust Acad Res Libr.

[52] Kroll S, Forsman R (2010). A slice of research life: information support for research in the United States [Internet].

[53] Groenewegen D (2017). Yesterday and today: reflecting on past practice to help build and strengthen the researcher partnership at Monash University. New Rev Acad Librariansh.

