# Supplementary material for: Needs assessment for improving library support for dentistry researchers
Source: J Med Libr Assoc. 2019 Jul 1;107(3):352–63. doi: 10.5195/jmla.2019.556 (PMC6579589; doi:10.5195/jmla.2019.556)
Supplement: Appendix [file jmla-107-352-s001.pdf]

Journal of the Medical Library Association

Supplemental content to
J Med Libr Assoc. Jul;107(3):dx.doi.org/10.5195/jmla.2019.556
www.jmla.mlanet.org
© He, Gerbig, Kirby 2019

Needs assessment for improving library support for dentistry researchers

Helen Yueping He; Madeline Gerbig; Sabrina Kirby

APPENDIX

Survey instrument

Welcome! The Dentistry Library is conducting a study in order to gain a better understanding of the needs profile of faculty researchers who use services at the Dentistry Library, with the ultimate goal of enhancing service provision at the Dentistry Library. We invite you to participate in this brief survey of approximately fifteen minutes. The insight afforded to the Dentistry Library through this research will allow more meaningful and efficient support delivery and will guide future service development in order to maximize the Dentistry Library's return on investment.

All survey responses are anonymous and confidential. Participation in this survey is completely voluntary. You may choose to leave the survey at any time, in which case your data will not be submitted or stored. Please note that once survey responses have been submitted, they cannot be withdrawn.

We thank you for your participation in improving service at the Dentistry Library!

This survey has been reviewed by the Research Ethics Board. For more information, please contact the researchers.

Demographic and general questions

1. For how many years have you have been actively involved in academic research?
   a. 0–5 years
   b. 6–10 years
   c. 11–20 years
   d. 20+ years

2. Which of the following best describes your primary research interest?
   a. Biomaterial and biomedical engineering
   b. Connective tissue and regenerative medicine
   c. Dental public health
   d. Education research
   e. Microbiology
   f. Oral pathology and cancer
   g. Pain and neuroscience
   h. Clinical research
   i. Other (please specify)

3. Are you interested in library workshops?
   If YES, q. 3a: What is the preferable format for library workshops?
   a. In person
   b. Videos
   c. Webinar
   d. Other (please specify)
   e. Not interested
   If NO, q. 3b: Why? (free form answer)

JMLA Journal of the Medical Library Association

SC
Supplemental content to
J Med Libr Assoc. Jul;107(3):dx.doi.org/10.5195/jmla.2019.556
www.jmla.mlanet.org
© He, Gerbig, Kirby 2019

4. Please rank the following services in order of usefulness. Assign the highest ranking to the service you feel would be most useful and the lowest ranking to the service you feel would be least useful.
   a. Information services for funding and grant application
   b. Grey literature searching assistance
   c. Research dissemination assistance
   d. Research impact assessment assistance

Information services for funding and grant applications

5. Please rank the following services based on how valuable you feel they would be to the funding/grant application process. Assign the highest ranking to the service you feel would be most valuable and the lowest ranking to the service you feel would be least valuable.
   a. Provision of general funder policy guidelines
   b. Literature search advice
   c. Reference management support
   d. Assistance with remaining current with research topics in your field
   e. Assistance with preparing impact statements for funders
   f. Assistance with preparing the data management plans for the grant application
   g. Assistance with open access requirements for funders
   h. Provision of guidelines for writing and/or publication
   i. Grant and funding databases training
   j. Assistance with identifying research priorities/known uncertainties
   k. Assistance with mentorship program with successful grant seekers

6. Which of the following digital resources/tools do you use (if any) when completing applications for funding or grants? Please select all that apply:

   Researcher profiling tools
   a. ORCID
   b. Researcher ID
   c. Scopus Author ID
   d. Google Scholar ID
   e. Other (please specify)

   Collaboration tools (share ideas, etc.)
   a. Google Drive
   b. Evernote
   c. Dropbox
   d. Trello
   e. Popplet
   f. Edmodo
   g. Pulse
   h. LabGuru
   i. Microsoft Teams
   j. Basecamp
   k. Slack
   l. MeisterTask (etc.)
   m. Other (please specify)

JMLA Journal of the Medical Library Association

Supplemental content to
J Med Libr Assoc. Jul;107(3):dx.doi.org/10.5195/jmla.2019.556
www.jmla.mlanet.org
© He, Gerbig, Kirby 2019

Reference management tools
a. EndNote
b. Refworks
c. Zotero
d. Mendeley
e. Other (please specify)

7. From where do you primarily receive your funding?
   a. Internal Faculty of Dentistry funding
   b. Tri-agency
   c. Public funding (i.e., municipal, provincial, or federal government funding)
   d. International funding
   e. Noncommercial third party (please specify)
   f. Industry
   g. Other (please specify)

Grey literature

The following questions address the topic of research requiring the use of grey literature. "Grey literature" refers broadly to the set of materials not published through traditional commercial and academic means (i.e., in academic journals), but that nevertheless have value as sources of information for scholarly research. Examples of such materials are government reports, conference proceedings, and theses/dissertations, among many others.

8. In a typical paper, what percentage of your citations come from sources that would be considered "grey literature," based on the above definition?
   a. 0%
   b. 1%–20%
   c. 21%–40%
   d. 41%–60%
   e. 61%–80%
   f. 81%–100%

9. How important do you consider grey literature to be to your research? Please rate based on the following scale:
   0=not important at all
   1=not very important
   2=neutral
   3=somewhat important
   4=very important

10. Rank the following varieties of grey literature from highest relevance to your research to lowest relevance.
   a. Theses and dissertations
   b. Conference and seminar proceedings
   c. Government publications
   d. Professional organization publications
   e. Research reports
   f. Other (please specify)
   g. None

JMLA Journal of the Medical Library Association

SC
Supplemental content to
J Med Libr Assoc. Jul;107(3):dx.doi.org/10.5195/jmla.2019.556
www.jmla.mlanet.org
© He, Gerbig, Kirby 2019

11. Which of the following resources do you use (if any) when searching grey literature? Please select all that apply.
    a. GreyNet
    b. OpenGrey
    c. OAIster
    d. Custom Google search for government documents (Canadian government docs, Canadian health departments and agencies, Ontario Public Health Units)
    e. Clinical trials databases
    f. Library catalogue
    g. Thesis/dissertation databases
    h. Databases that index conference proceedings
    i. Institutional repositories
    j. Associations/institutions website
    k. Journal website
    l. Conference website
    m. Search engine (e.g., Google, Yahoo)
    n. Colleagues
    o. Other (please specify)
    p. None of the above

12. Please rate the following statement based on the scale below: "The grey literature for my field and/or area of research is easy to search and access."
    0=disagree strongly
    1=disagree somewhat
    2=neutral
    3=agree somewhat
    4=agree strongly

Research dissemination

13. Please rank the following services based on your level of need during the publication process. Assign the highest ranking to the area in which you have the greatest need and the lowest ranking to the area in which you have the least need.
    a. Assistance with meeting funder mandates and/or requirements
    b. Assistance with negotiating licenses
    c. Assistance with copyright issues
    d. Assistance with identifying publication venues
    e. Assistance with reference management
    f. Assistance with open access publication
    g. Assistance with archiving publications
    h. Other (please specify)

14. Please indicate your current engagement with open access publication.
    a. I am not familiar with open access publishing models
    b. I am familiar with open access publishing models but do not employ them in my own research
    c. I occasionally use open access publishing models for my own research
    d. I regularly use open access publishing models for my own research

Supplemental content to
J Med Libr Assoc. Jul;107(3):dx.doi.org/10.5195/jmla.2019.556
www.jmla.mlanet.org
© He, Gerbig, Kirby 2019

15. What journal characteristic is most important to you when you are deciding where you should publish an article?
    a. The journal is an open access journal
    b. The journal permits scholars to publish articles for free
    c. The journal permits scholars to retain copyright
    d. The journal is circulated widely and accessible to readers not only in developed nations, but also in developing nations
    e. The journal has a high impact factor
    f. The journal has an excellent academic reputation
    g. The journal's area of coverage is very close to my immediate area of research
    h. The journal is indexed by major indexers and abstractors
    i. If accepted, the journal will publish my article quickly, with relatively little delay

16. More and more researchers are sharing their work in nontraditional ways online. Are you using any of the following new publication modes or venues? Please select all that apply.

    Researcher networks
    a. CoS
    b. Google Scholar
    c. Mendeley
    d. Academia.edu
    e. ResearchGate

    Free online services for sharing content
    a. SlideShare
    b. Figshare
    c. Vimeo
    d. Vine

    Institutional/personal social media platform
    a. Personal web page
    b. LinkedIn
    c. Blog
    d. YouTube
    e. Twitter
    f. Facebook

Research impact assessment

17. Please rank the following services based on how valuable you feel they would be to the research impact assessment process. Assign the highest ranking to the service you feel would be most valuable and the lowest ranking to the service you feel would be least valuable.
    a. Research metrics training
    b. Citation analysis guidance
    c. Assistance with tenure and promotion profiles
    d. Assistance with identifying collaborators
    e. Assistance with identifying funding agencies
    f. Assistance with topical bibliometric analysis
    g. Provision of research trend reports

JMLA Journal of the Medical Library Association

SC
Supplemental content to
J Med Libr Assoc. Jul;107(3):dx.doi.org/10.5195/jmla.2019.556
www.jmla.mlanet.org
© He, Gerbig, Kirby 2019

h. Assistance with benchmarking at departmental and institution level
i. Altmetrics training
j. Altmetrics support
k. One-on-one consultations
l. Other (please specify)

18. Which of the following resources do you use (if any) when conducting research impact measurement activities? Please select all that apply.
    a. Scopus
    b. SciVal
    c. Web of Science
    d. InCite
    e. Google Scholar Metrics
    f. Essential Science Indicators
    g. Publish or Perish
    h. Altmetrics
    i. Impact Story
    j. Other (please specify)

19. With which one of the following areas of research impact assessment do you feel you could most benefit from library support?
    a. Research output metrics (e.g., number of publications, citation count, h-index)
    b. Journal-based metrics (e.g., impact factor)
    c. Alternative metrics (e.g., social media presence)
    d. Metric visualization
    e. Qualitative analysis
    f. Other (please specify)
    g. None

20. Are you currently using any of the following strategies to maximize your research impact?
    a. Strategic publishing (e.g., careful selection of journals for the publication of your work)
    b. Use of open access publication models
    c. The submission of your publications to an institutional repository such as the University of Toronto's TSpace
    d. Use of multiple channels to share your work
    e. Other (please specify)

This is the end of the survey. We thank you for your time and participation. You are invited to provide an email address at which you can be contacted in order to participate in a focus group interview with other interested respondents. This focus group is completely voluntary: you do not need to provide an email and participate in the focus group in order to provide survey responses. Any contact information provided will be kept confidential and stored separately from survey responses.

You may withdraw from this survey at any time before hitting the submission button. Please note, once you submit your answers they cannot be withdrawn from the study. All data will be anonymized in published results. Please click the "Submit" button to submit your completed survey.
